# Electroacupuncture Zusanli (ST36) on Release of Nitric Oxide in the Gracile Nucleus and Improvement of Sensory Neuropathies in Zucker Diabetic Fatty Rats

**DOI:** 10.1093/ecam/nep103

**Published:** 2011-06-08

**Authors:** Pei-Jing Rong, Sheng-Xing Ma

**Affiliations:** Department of Obstetrics and Gynecology, David Geffen School of Medicine at University of California Los Angeles, Harbor-UCLA Medical Center, Torrance, CA 90502, USA

## Abstract

The purpose of these studies was to examine the effects of electroacupuncture (EA) Zusanli (ST36) on release of nitric oxide (NO) in the gracile nucleus (GN) and determine if functional neuropathic changes were modified by EA ST36-induced NO in the nucleus in Zucker diabetic fatty (ZDF) rats. The foot withdrawal responses to mechanical, thermal and cold stimuli were measured before and after EA stimulation. A microdialysis probe was implanted in the GN and dialysate samples were collected 20 min before, during and after EA ST36. Total nitrate and nitrite (NO_*x*_^*−*^) concentrations in the samples were quantified by using chemiluminescence. The baseline dialysate NO_*x*_^*−*^ concentrations in the GN were decreased in ZDF rats compared to lean control (LC) rats (*P* < .05). In ZDF rats, dialysate NO_*x*_^*−*^ releases in the GN were markedly increased during EA ST36, whereas in LC rats, the releases were moderately enhanced at 20–40 min after EA ST36. The withdrawal latencies to mechanical, cold and thermal stimuli were significantly improved 20 min after EA ST36 both in LC and ZDF rats, but not altered by non-acupoint stimulation. The withdrawal latencies to EA ST36 were further potentiated by 3-morpholinyl-sydnoneimine and inhibited by N^G^-Propyl-l-arginine infused into the GN in ZDF rats (*P* < .05). These results show that EA ST36 increases NO release in the GN, and NO in the nucleus modifies withdrawal latencies to mechanical, cold, and thermal nociception stimuli. Data suggest that EA ST36 induces NO release in the GN, which contributes to improvement of sensory neuropathies in rats.

## 1. Introduction

Sensory neuropathy is a frequent complication of diabetes that is accompanied with pain, paresthesia and reduced temperature and vibration perception thresholds [1, 2]. Previous studies using the Zucker diabetic fatty (ZDF) rat model have shown that nerve conduction velocity is decreased [3–5] and pain/pressure thresholds are abnormal at 11–13 weeks of age in diabetic versus lean rats [6–8]. The treatment of sensory neuropathy of diabetes has become a challenge especially in the long-term management of neuropathic pain [9, 10]. Consequently, diverse treatments are used for diabetic neuropathy, including non-invasive drug therapies (antidepressants, antiepileptic drugs and membrane stabilizing drugs), invasive therapies (nerve blocks, ablative surgery) and alternative therapies (e.g., acupuncture and herbal) [10–13]. No consistently effective treatment for diabetic neuropathy is available and patients are forced to struggle with medications that provide only partial relief [9, 14]. Acupuncture and electroacupuncture (EA) is widely applied to treat various diseases including diabetic neuropathies and is becoming more recognized worldwide. Several clinical studies suggest that acupuncture decreases neuropathy-associated pain and improves nerve sensation [14–16]. Although acupuncture is a safe and effective therapy for improvement in symptoms of sensory neuropathy, the number of long-term pain relief is small and the therapeutic effects need to be improved [14, 15].

**Figure 1:**
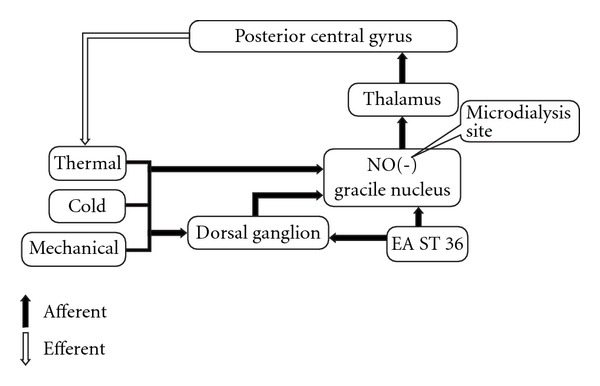
A schematic model of signal transduction of somatosensory information and acupuncture signals in the dorsal funiculus tract brain circuit. Acupuncture stimulation of hindlimb acupoint, ST36, induces NO release in the GN, which causes NO-mediated inhibition of mechanical, cold and thermal stimuli through afferents-dorsal medulla-thalamic pathways.

Studies on the mechanisms of action have revealed that endogenous opioid peptides in the central nervous system play an essential role in mediating the analgesic effect of EA [17]. Chang et al. [18] suggest that EA stimulation at the Zhongwan acupoint induces secretion of endogenous beta-endorphin that reduces plasma glucose concentration in an insulin-dependent manner. Another opinion shows that nitric oxide (NO) mediates acupuncture signals through the dorsal medulla-thalamic pathways [19], as illustrated in Figure 1. The gracile nucleus (GN) is the site in the dorsal medulla that receives primary sensory afferents projecting from the hindlimb [20, 21]. A number of recent studies have suggested that the GN is an important area responsible for sensory/pain regulation [22–24]. Previous studies from this laboratory have demonstrated that expression of neuronal NO synthase (nNOS) in the GN is induced by sciatic nerve injury and EA stimulation of hindlimb acupoints in rats [25–27]. In SD rats, EA stimulation of Zusanli (ST36), a vital acupoint on the leg, decreased arterial blood pressure and the effects were facilitated by a microinjection of an exogenous NO donor in the GN [28]. NO is one of the most important messenger molecules produced in many cell types, including the neurons in the central nervous system (CNS) [29, 30]. It has been demonstrated that neuronal NO generation is impaired and is associated with hyperalgesia in diabetic rats [30, 31]. However, the mechanisms and actions of NO release responsible for the analgesic effects of EA are not understood.

The purpose of this study is to examine the hypothesis that there is a neural circuit related to transduction of somatosensory information and EA ST36 in the afferent inputs-dorsal medulla-thalamic-efferent pathways (Figure 1). EA stimulation of ST36 induces NO release in the GN resulting in inhibition of heat, cold and mechanical sensitivities through the dorsal funiculus tract brain circuit. The relationship between medullar NO releases and sensory neuropathies was determined by quantification of dialysate NO metabolites in the GN and the foot withdrawal responses to mechanical, thermal and cold stimuli in ZDF compared to age-matched lean control (LC) rats. The effects of EA ST36 on NO release in the GN and improvements in hyperalgesia to thermal, cold and tactile stimulations were quantified before and after EA stimulation of ST36 and non-acupoint in ZDF rats. In order to investigate the direct effects of NO presence in the GN on sensory neuropathic changes, withdrawal latencies to mechanical, thermal and cold stimuli were measured before and after microinfusion of 3-morpholinyl-sydnoneimine (SIN-1, an exogenous NO donor) or N^G^-Propyl-l-arginine (NPLA, a selective inhibitor of neuronal NO synthesis) into the GN in ZDF rats.

## 2. Methods

### 2.1. Experimental Animals

Experiments were performed on ZDF rats (11–12 weeks) and age-matched LC rats (Genetic Models, Inc., Indianapolis). ZDF rats were fed Purina 5008 (16.7 kcal% of fat) diet chow [7]. All rats were given continuous access to food and water. The protocol was approved by the Harbor-UCLA Animal Use Committee and was in accord with the American Association for the Accreditation of Laboratory Animal Care (AAALAC) and National Institutes of Health (NIH) guidelines. Animals were maintained on a 12-h light-dark cycle in temperature and humidity-controlled rooms. The body weight and blood glucose concentrations were assessed before and at the end of the treatments. The concentration of blood glucose was measured with a glucometer (Elite, Bayer), and the blood sample was obtained by performing a small incision at the tail end.

### 2.2. Measurements of Tactile Withdrawal Latency

The paw withdraw latency to mechanical nociceptive stimulation was elicited by applying a von Frey filament (Semmes-Weinstein Von Frey Aesthesiometer Kit, Cimerio, Italy) as previously described [32, 33]. The rats were placed in a cage with wire mesh floor and allowed to habituate for 15 min before each measurement. A paw withdrawal response was elicited by applying a 29 g von Frey filament on the planter surface of the ipsilateral hind paw. A positive response was indicated by a sharp withdrawal of the paw. The force applied to elicit a reflex removal of the ipsilateral hind paw was monitored. The authors chose a 29 g von Frey filament according to previous report [33] and our preliminary study revealed that more than half of applying a 29 g von Frey filament induce withdrawal responses in the LC rats. The withdrawal latencies were expressed in seconds, which was the time the rat withdrew its hind foot in reaction to the stimulation. The inter-stimulus interval is 5 min. The decimal was 0.1 s, and four latencies were recorded and averaged for the ipsilateral hind paw in each test session.

### 2.3. Measurements of Thermal and Cold Withdrawal Latencies

Withdrawal latencies of the hind foot to cold and thermal stimuli were tested using a plate kept at 4°C and 52°C, respectively. The plate tool was applied to the dorsal surface of the hind foot once in each trial, for four trials [32–35]. The withdrawal latencies were expressed in seconds, which was the time the rat withdraw its hind foot in reaction to the stimulation. The heat or cold source created a constant stimulus until the rats voluntarily withdrew their paw. The cut time was 10 s to avoid injury to the skin. The decimal was 0.1 s and four measurements were averaged for each paw. Testing was repeated every 5 min for a total of four trials in each test session.

### 2.4. EA Stimulation

The rats in the experiments were placed in the immobilization apparatus consisting of a plastic cylinder. EA stimulation was bilaterally applied to the points of Zusanli (ST36) at the depression below the knee from the anterior crest of the tibia [28, 36]. As a control for the specific acupoint effects, stimulation was also applied to the “non-acupoints” located nearby ST36 in the hamstring muscles as described [28, 37]. The needle electrodes (27-gauge sharpened stainless-steel insect pins) were inserted percutaneously into a depth of 4 mm at the points of ST36 as previously described [28, 36]. EA stimulation was performed using a battery-powered stimulator (Skylark Electro-stimulator SK-700B) connected to each pair of needle electrodes. Biphasic pulse electrical stimuli were applied to the acupoints using a current 1.0 mA with duration of 1.0 ms for 20 min in conscious rats. Different frequencies (3, 10, 30 and 60 Hz) of EA were applied daily at ST36 or at non-acupoint in ZDF and LC rats for 5 days. The mechanical tolerance and withdrawal threshold of the foot in responses to EA ST36 were measured at 10-min intervals before, and 10 and 30 min after each phase of EA stimulation.

### 2.5. Microdialysis in the GN

The objective was to determine whether NO releases paralleled changes in mechanical threshold and latencies of foot withdrawal to noxious heat or cold stimulation induced by EA ST36. The rats were placed in a stereotaxic apparatus with the head flexed at 45° to facilitate exposure of the obex under anesthesia with ketamine (100 mg kg^−1^) and xylazine (13 mg kg^−1^, i.p.). A heating pad was used to prevent heat loss and maintain body temperature at 37.5°C. A CMA 12 microdialysis siliconized guide (CMA/Microdialysis, North Chelmsford, MA, USA) was implanted into the GN using previously described methods [38, 39]. The guide cannula was secured in place with dental cement. The animals were allowed to recover. A microdialysis probe (CMA/11, the cuprophane dialysis membrane, length 1 mm, diameter 0.24 mm, molecular cut-off 6 kDa; CMA/Microdialysis, North Chelmsford, MA, USA) was inserted into the GN (1 mm under the surface) through the cannula 48 h after surgery. Artificial cerebrospinal fluid (pH adjusted to 7.4 CMA/Microdialysis) was infused at a rate of 3 *μ*l min^−1^. The inlet/outlet dialysis capillaries were connected to polyethylene tubing. The inlet tube was connected to a 50 *μ*l microsyringe driven by the CAM 102 microdialysis pump. The outlet was positioned in a collecting tube set in the ice

To verify the site of microdialysis, 2% pontamine sky blue was injected into the GN after all the experiment procedures had been done. The rats were deeply anesthetized; the brains were removed and stored in a 10% paraformaldehyde solution. The frozen brain tissue was sectioned in the coronal plane (40 *μ*m). The site of the dialysis tube was verified histologically in the brain sections after cresyl violet staining [40]. The blue stained area in the brainstem containing the GN was countered under the light microscope. The results from the animals with injection diffusing out of the GN were excluded from statistic data.

#### 2.5.1. Biochemical Analysis of NO_*x*_^*−*^ Concentrations

The concentrations of NO_*x*_^*−*^ (NO_2_^*−*^ and NO_3_^*−*^) in the dialysate samples were measured using an ozone phase chemiluminescence (NOA280i, GE Analytical Instruments, Boulder, CO, USA) as described [38, 41]. The measurement was conducted in a blinded manner. Briefly, 5 *μ*l dialysate samples (fresh or previously frozen) were reduced using a Vanadium (III)/HCl solution. The nitrate calibration curve was established using known concentrations of NaNO_3_ dissolved in the sterile nitrogen-free water. The total amount of NO_3_^*−*^ in each dialysate sample was calculated by integration of the signal peaks using the nitrate calibration curve. All samples were measured in duplicate. The presence of (NO_2_^*−*^) in our dialysate samples was close to the water basal level. Therefore, the final NO_*x*_^*−*^ concentration in the dialysate was expressed in micromolars with no allowance made for NO_2_^*−*^. The minimum sensitivity level of NO amount is 1.0 pmol.

### 2.6. Experimental Protocol

The rats were divided into two groups (*n* = 20), LC rats group (*n* = 10) and ZDF rats group (*n* = 10). In each group, EA stimulation of ST36 and non-acupoint was performed using 1.0 mA, duration of 1.0 ms for 20 min. These stimulation parameters with 3, 10, 30 or 60 Hz were randomly conducted at day 1, 2, 3 and 4, respectively. Measurements of mechanical, thermal and cold hyperalgesia were conducted twice at 20-min intervals before (averaged to obtain a control value) and at 20, 40, 60, 80, 100 and 120 min after EA stimulation in conscious rats.

On the fifth day, EA stimulation was performed using 10 Hz based on the results from Day 1 to 4 tests. Artificial cerebral spinal fluid was perfused in the unilateral side of the GN. The dialysate samples were collected during EA and every 20 min after EA for a total of 120 min for measurements of NO_*x*_^*−*^ concentrations. After a 20-min period of dialysis equilibration, 20-min dialysate samples were collected during each subsequent perfusion condition. In each treatment group, the dialysate samples were collected at two 20-min intervals before stimulation (averaged to obtain a control value), 20-min interval during EA stimulation and four 20-min intervals after the stimulation. In another group of rats, SIN-I (10 *μ*M) or NPLA (100 *μ*M) was dissolved in the perfusion fluid and infused via retrograde microdialysis into the nucleus for 20 min. Then the measurements of mechanical, thermal and cold hyperalgesia were conducted before and after EA ST36 or EA non-point.

At the end of the experiment, the probe was checked for the presence of air bubbles, and the position in the GN was verified by microscopy of perfusion-fixed sections [39, 40]. Following completion of the experiments, the rats were sacrificed by sodium pentobarbital (150 mg kg^−1^, i.v.). The brains were removed and stored in a 10% paraformaldehyde solution. The site of microdialysis was verified histologically on coronal and sagittal brain sections after crystal violet staining. Serial coronal sections of 40-*μ*m thickness were made using a freezing microtome (Microm-HM400). Histological verification was carried out and referenced to the rat brain in stereotaxic coordinates [42]. The microdialysis area in the brainstem containing the GN was examined under the light microscope. The results from the animals with infusions diffusing out of the nucleus and incorrect infusion site were excluded from statistic data.

### 2.7. Chemicals

The chemicals used in these experiments were NPLA (Calbilchem) and SIN-1 (Tocris).

### 2.8. Statistical Analysis

The results were expressed as mean ± standard error mean (SEM). LC and ZDF rats were used for each defined group. Analysis of variance (one-way ANOVA and Turkey HSD) and Student's *t*-test (unpaired) were used to analyze significant difference using software SSPS 11.5 (SSPS Inc., Chicago, IL, USA). A *P*-value < .05 was considered significant.

## 3. Results

Of the 20 experimental rats, the body weights were of minimal variation, 509.1 ± 46.3 g in ZDF rats versus 346.2 ± 9.3 g in LC rats (*P* < .01, *n* = 10/group). Blood glucose was markedly elevated (*P* < .001) in ZDF rats 301.3 ± 25.1 versus LC rats 122.1 ± 4.3 mg/dl^−1^.

### 3.1. Behavior Determination of Neuropathies In Vivo

**Figure 2:**
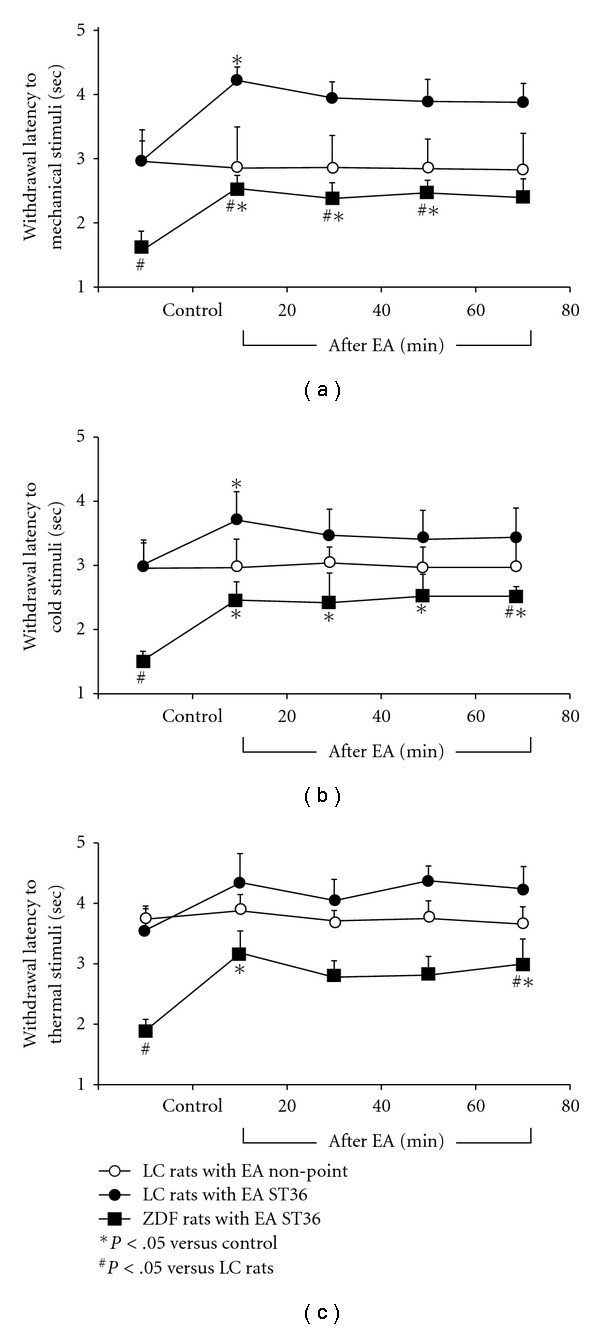
Withdrawal latencies to mechanical (a), cold (b) and thermal stimuli (c) on foot following EA stimulation of ST36 in conscious ZDF rats compared to LC rats. Values were expressed as mean ± SEM (*n* = 10). *P < .05 versus non-point control, ^#^ *P* < .05 versus LC rats.

The withdrawal latencies to the application of mechanical, thermal or cold stimulation were examined in ZDF rats compared to age-matched LC rats (*n* = 10 in each group). The baseline withdrawal latencies to mechanical stimulation on the foot were significantly decreased (*P* < .05) in ZDF versus LC rats (Figure 2(a)). The withdrawal latencies to cold stimuli of ZDF rats were also reduced compared to LC rats (*P* < .05) (Figure 2(b)). The withdrawal latencies to thermal stimuli of ZDF rats were markedly reduced compared to LC rats (*P* < .05), as shown in Figure 2(c).

The withdrawal latencies to application of mechanical or cold stimulation were increased 20 min after EA ST36 in both LC and ZDF rats (*P* < .05), as shown in Figures 2(a) and 2(b). The values of the withdrawal latencies to thermal stimuli tended to be high 20 min after EA ST36 in LC rats compared to control, but this increase fell short of statistical significance. The withdrawal latencies to thermal stimuli were significantly increased 20 min after EA ST36 in ZDF rats (Figure 2(c)). One-way ANOVA and Turkey HSD analysis indicated that there were no significant differences of withdrawal latencies to cold and thermal stimuli in ZDF versus LC rats after EA ST36. In contrast, EA stimulation of non-acupoint did not induce detectable changes in withdrawal latencies to mechanical, cold and thermal stimuli in LC rats.

### 3.2. Withdrawal Latencies to EA Stimulation of ST36 with Different Frequency

**Figure 3:**
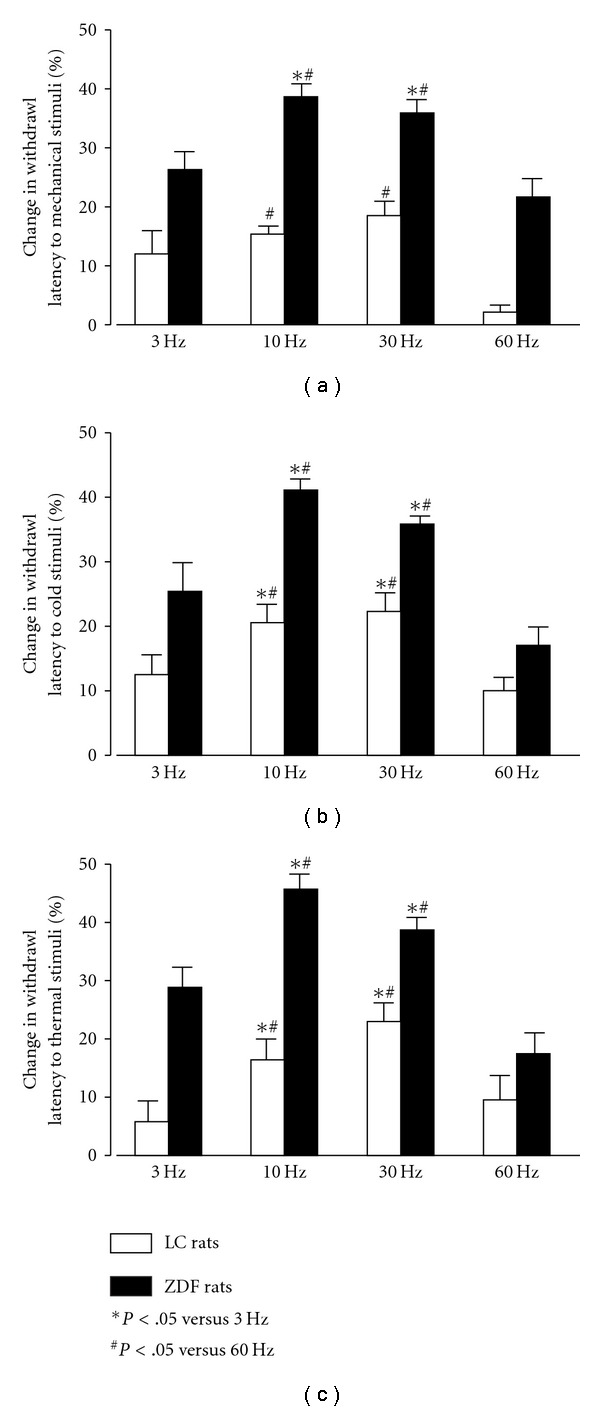
Percentage changes in withdrawal latencies to mechanical (a), cold (b) and thermal stimuli (c) induced by different frequency EA ST36 in conscious LC rats and ZDF rats. Parameters of stimulation: 6 V, 1 ms pulse duration, 3, 10, 30 and 60 Hz for 20 min. ANOVA revealed significant differences of 10 and 30 Hz compared with 3 and 60 Hz EA stimulation (*P* < .05, *n* = 10/group).

The effects of different frequencies on sensory neuropathic changes were studied following EA ST36 using 3, 10, 30 and 60 Hz in ZDF versus LC rats. Withdrawal latencies to mechanical, thermal or cold stimulation were continually observed for 3 days and the averaged numbers were used to serve as control values before EA. Following EA ST36 with constant voltage and duration in LC rats, the withdrawal latency responses were enhanced on the frequency from 3 to 30 Hz, but did not increase further at 60 Hz (Figure 3). In ZDF rats, the values of increases in withdrawal latency responses to 10 and 30 Hz EA ST36 were better than those induced by 3 Hz and 60 Hz, as shown in Figure 3. However, the withdrawal latencies to EA ST36 were significantly attenuated in 60 Hz compared with 10 and 30 Hz EAs in both ZDF and LC rats (*P* < .05). There was no statistically significant difference between 10 and 30 Hz EA ST36 (Figure 3).

### 3.3. Baseline of the Dialysate NO_*x*_^*−*^ Concentrations in the GN of LC Rats and ZDF Rats

**Figure 4:**
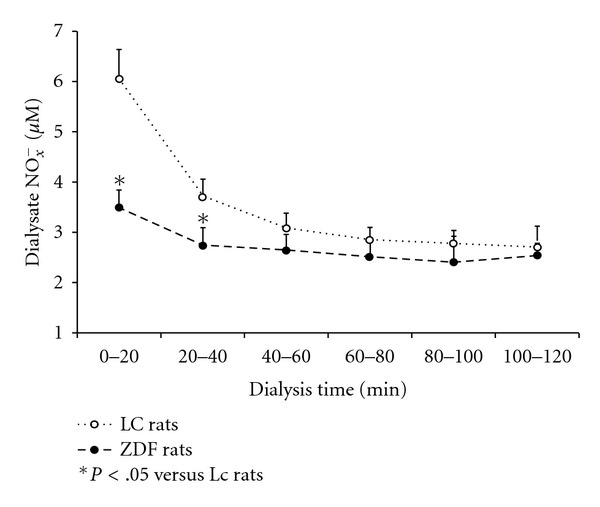
The time intervals of dialysate nitrite plus nitrate (NO_*x*_^*−*^) concentrations in the GN in ZDF rats versus LC rats. Dialysate NO_*x*_^*−*^ from perfusion periods was analyzed as follows: 0–20, 20–40, 40–60, 60–80, 80–100 and 100–120 min. Each point represents mean ± SEM (*n* = 10). *P < .05 versus LC rats. There was a significant difference of the dialysate NO_*x*_^*−*^ concentrations between ZDF rats and LC rats in 0–20 and 20–40 min (*P* < .05).

NO metabolites, total nitrate and nitrite (NO_*x*_^*−*^) concentrations in the dialysate samples were collected at 20-min intervals obtaining at 20, 40, 60, 80, 100 and 120 min in ZDF and LC rats (Figure 4). In ZDF rats, baseline dialysate NO_*x*_^*−*^ releases in the GN were significantly lower than those in LC rats during the first and second 20-min intervals of dialysis (*P* < .05). The levels of dialysate NO_*x*_^*−*^ concentrations at 40–60, 60–80 and 80–100 min in ZDF rats suggested a slight reduction compared to LC rats. There were similar dialysate NO_*x*_^*−*^ concentrations at 100–120 min between ZDF rats and LC rats, as shown in Figure 4. In LC rats, baseline dialysate NO_*x*_^*−*^ concentration during the first 20-min interval of dialysis was much higher than those in ZDF rats (Figure 4). Dialysate NO_*x*_^*−*^ concentrations were markedly reduced at 40–120 min dialysis compared to 0–40 dialysis in LC rats (*P* < .05). However, there were moderate reductions of dialysate NO_*x*_^*−*^ concentrations at 40–120 min dialysis compared to 0–20 dialysis in ZDF rats, as shown in Figure 4.

### 3.4. Responses of Dialysate NO_*x*_^*−*^ Releases in the GN to EA ST36

**Figure 5:**
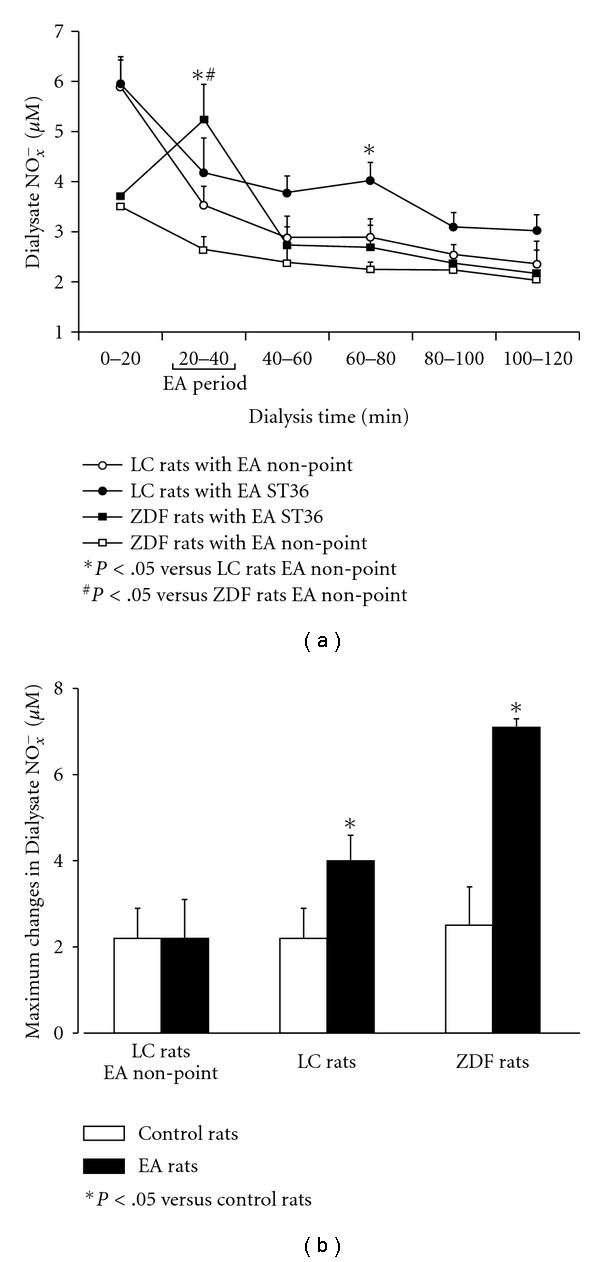
Time response curves of changes in dialysate NO_*x*_^*−*^ concentrations in the GN following EA stimulation of ST36 and non-acupoint in ZDF and LC rats during 120 min (a). Maximum changes in dialysate NO_*x*_^*−*^ releases from the GN induced by EA ST36 in LC rats and ZDF rats were calculated by comparing the difference of the highest NO value in EA groups and the basal NO_*x*_^*−*^ value at that time in control groups without the stimulation (b). Each point represents mean ± SEM (*n* = 10).

Figure 5(a) shows dialysate NO_*x*_^*−*^ concentrations in the GN before, during and 20, 40, 60 and 80 min after EA stimulation of ST36 and non-acupoint in ZDF and LC rats. There was no significant change in NO_*x*_^*−*^ concentrations following EA non-acupoint in both ZDF and LC groups (Figure 5(a)). However, NO_*x*_^*−*^ concentrations in the GN of ZDF rats were markedly increased at 20–40 min during EA ST36 (*P* < .05, *n* = 8). In LC rats with EA ST36, dialysate NO_*x*_^*−*^ concentrations in the GN were marginally increased 20 min (*P* = .086) and significantly increased 40 min after EA (*P* < .05), as shown in Figure 5(a). Dialysate NO_*x*_^*−*^ concentrations in the GN were significantly increased during EA ST36 in both LC and ZDF dialysis groups compared to the control without EA groups (Figure 5(a)). 

The maximum changes in dialysate NO_*x*_^*−*^ collected from the GN induced by EA ST36 in LC rats and ZDF rats were compared with their control groups without the stimulation (Figure 5(b)). The EA stimulation was given at ST36 (*n* = 8) or non-point (*n* = 8) for 20 min. The dialysate was collected in the GN every 20 min for a period of 2 h (total of six collections). The maximum NO_*x*_^*−*^ change for each subject was determined by the highest NO_*x*_^*−*^ amount during the 2 h after EA stimulation. The change of the NO_*x*_^*−*^ concentration (*μ*M) was calculated by comparing the difference of the highest NO value in EA groups and the basal NO_*x*_^*−*^ value at that time in control groups. In LC rats, EA stimulation of non-acupoint did not induce detectable changes in maximum dialysate NO_*x*_^*−*^ concentration (Figure 5(b)). There were statistically significant differences in the maximum change of NO_*x*_^*−*^ releases between EA ST36 and control group without EA in LC rats (*P* < .05). In ZDF rats, maximum change in NO_*x*_^*−*^ release was markedly increased in the EA ST36 group compared to control rats without EA (*P* < .05), as shown in Figure 5(b).

### 3.5. Effects of an Exogenous NO Donor and an Inhibitor of Neuronal NO Synthesis in the GN on Withdrawal Latencies to EA ST36

**Figure 6:**
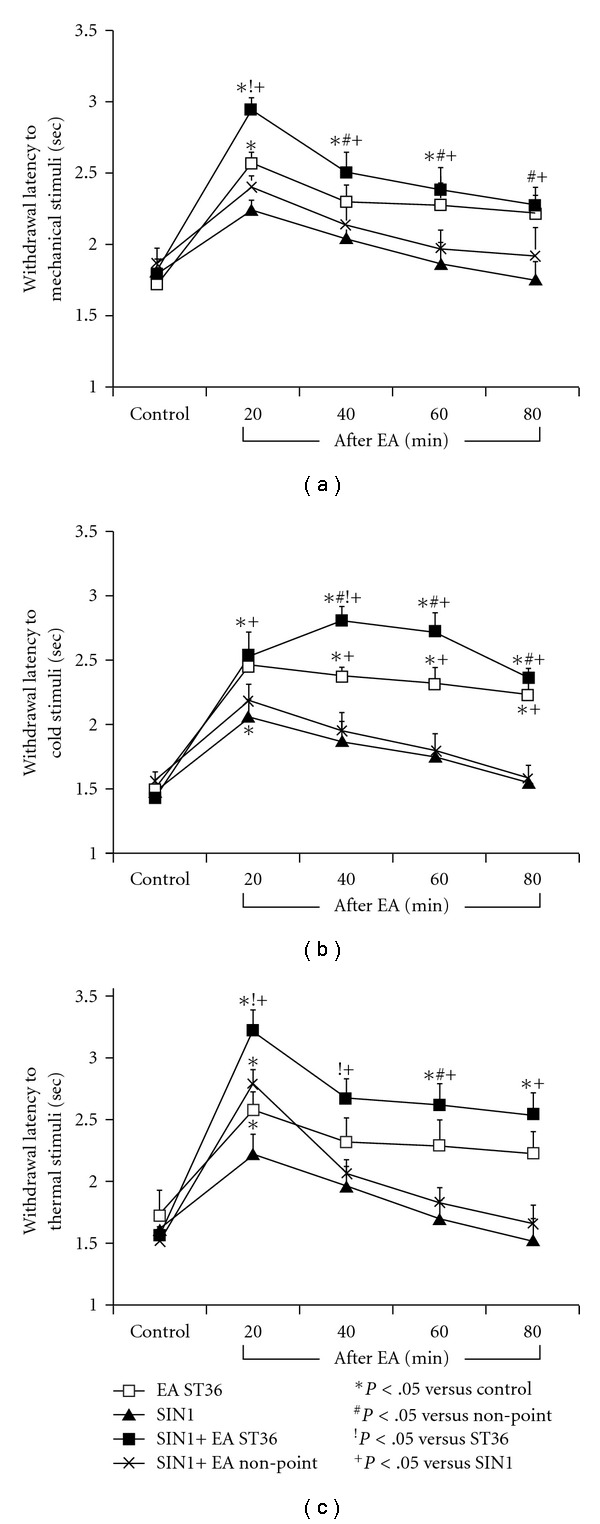
Changes in withdrawal responses of latency to mechanical, cold and thermal stimuli in ZDF rats following EA ST36 or non-acupoint with or without microinfusion SIN1 into GN. Values were expressed as mean ± SEM (*n* = 10). One-way ANOVA analysis indicated that there were significant differences between SIN1 infusion alone and SIN1 infusion plus EA ST36, and between EA ST36 and EA non-point following SIN1 infusion. Withdrawal latencies to mechanical stimuli (a) and thermal stimuli (c) were significantly increased at 20 min after EA ST36 plus SIN1 (*P* < .05). Withdrawal latencies to cold stimuli were increased at 40 min after EA ST36 (b).

Withdrawal latencies to mechanical, cold and thermal stimuli in ZDF rats were examined following EA ST36 or non-acupoint with or without microinfusion SIN1, an exogenous NO donor, into the GN.  Figure 6 shows that withdrawal latencies to mechanical stimuli (Figure 6(a)), cold stimuli (Figure 6(b)) and thermal stimuli (Figure 6(c)) were moderately enhanced at 20 min following SIN1 infusion alone in ZDF rats. However, withdrawal latencies to mechanic, cold and thermal stimuli were markedly increased by SIN1 infusion plus EA ST36 compare to SIN1 infusion alone. Withdrawal latencies to mechanical stimuli (Figure 6(a)) and thermal stimuli (Figure 6(c)) were significantly increased by SIN1 infusion plus EA ST36 compare to EA ST36 alone at 20 min after EA ST36, as shown in Figure 6. For the withdrawal latencies to cold stimuli (Figure 6(b)), there were significant differences between EA ST36 plus SIN1 infusion and EA ST36 alone at 40 min after EA ST36 (*P* < .05). One-way ANOVA analysis indicated that the changes in withdrawal latencies were significant differences between SIN1 infusion alone and SIN1 infusion plus EA ST36, and between EA ST36 and EA non-point following SIN1 infusion (Figure 6).

**Figure 7:**
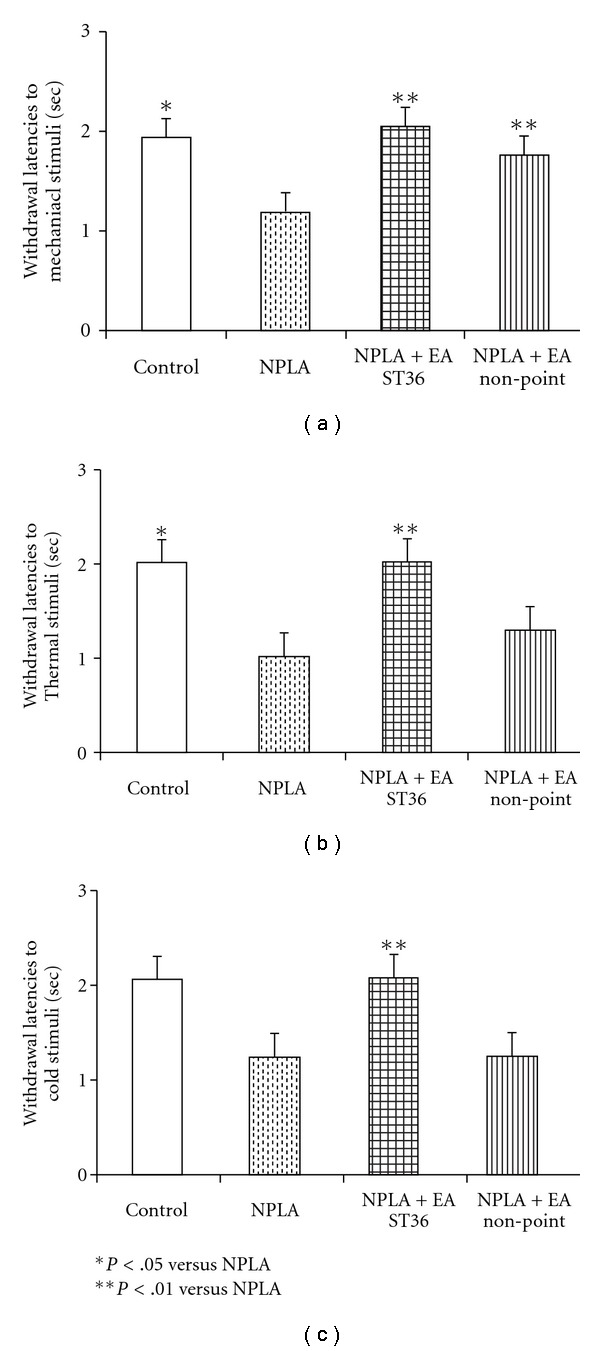
Withdrawal latencies to mechanical (a), cold (b), and thermal stimuli (c) on foot in ZDF rats following EA ST36 or non-acupoint with or without microinfusion NPLA into GN. Values were mean ± SEM (*n* = 5). *P < .05 versus control.

Withdrawal latencies to mechanical, cold and thermal stimuli in ZDF rats were tested before and after NPLA microinfusion, an selective inhibitor of neuronal NO synthesis into the GN.  Figure 7 shows that withdrawal latencies to mechanical stimuli (Figure 7(a)), cold stimuli (Figure 7(b)) and thermal stimuli (Figure 7(c)) were significantly decreased after microinfusion of NPLA into the GN. Following microinfusion of NPLA into the GN, withdrawal latencies to mechanical, cold and thermal stimuli in ZDF rats were not altered following EA stimulation of either ST36 or non-acupoint, as shown in Figure 7.

**Figure 8:**
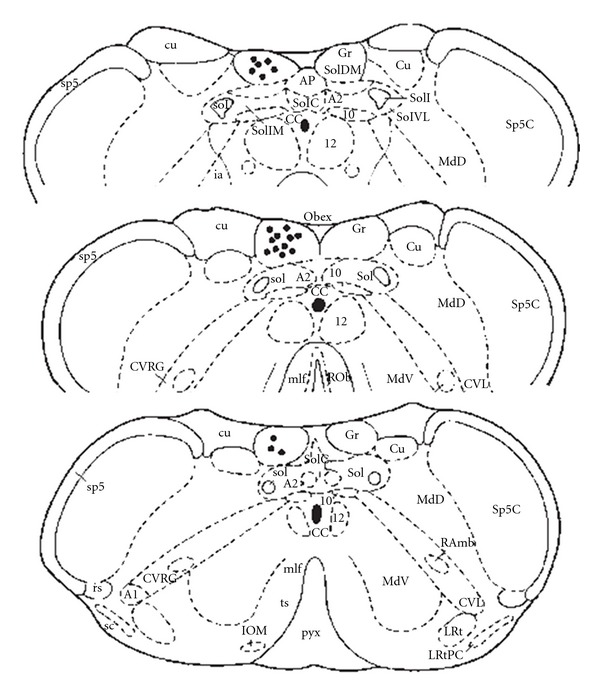
Illustration of verified the sites of microdialysis in the GN by coronal section of the dorsal medulla. Closed circle indicated the microdialysis tips in the gracile nucleus.

Figure 8 presents a medullary coronal section summarizing the locations of the GN sites for microdialysis during these studies. 

## 4. Discussion

We compared the foot withdrawal responses to mechanical, thermal and cold stimuli in ZDF and LC rats following EA stimulation of ST36. The relationship between effects of EA ST36 and NO releases in the GN were examined using microdialysis, and the effects of NO on withdrawal responses to mechanical, thermal and cold stimuli were observed following microinfusion of an exogenous NO donor and an inhibitor of neuronal NO synthesis into the GN. The major findings in these studies are (i) The withdrawal latencies to mechanical, cold and thermal stimuli were significantly reduced in ZDF rats compared with LC rats; (ii) EA ST36 with 10 and 30 Hz produced better effects on increases in withdrawal latencies to mechanical, cold and thermal stimuli compared to the stimulation with 3 and 60 Hz; (iii) The baseline dialysate NO_*x*_^*−*^ concentrations in the GN were decreased in ZDF rats compared to LC rats; (vi) Dialysate NO_*x*_^*−*^ releases in the GN were markedly increased during EA ST36 in ZDF rats and moderately enhanced 20–40 min after EA ST36 in LC rats; and (v) EA ST36-induced increases in withdrawal latencies to mechanical, cold and thermal stimuli were potentiated by microinfusion of an NO donor and inhibited by an inhibitor of neuronal NO synthesis into the GN in ZDF rats. The present study is the first evidence showing that dialysate NO_*x*_^*−*^ releases in the GN were markedly increased during EA ST36 in ZDF rats, while the gracile NO_*x*_^*−*^ releases were moderately enhanced 20–40 min after the EA stimulation in LC rats. Withdrawal latencies to mechanical, hot and cold stimuli in ZDF rats and LC rats were significantly increased following EA ST36 but were not altered by EA stimulation of non-acupoint. The results also demonstrate that withdrawal latencies to mechanical, hot and cold stimuli following EA ST36 were significantly potentiated by microinfusion of SIN1, an NO donor, into the GN and inhibited by microinfusion of NPLA, an inhibitor of neuronal NO synthesis into the nucleus in ZDF rats. The studies suggest that EA ST36 increases the release of NO in the GN associated with improvement of functional neuropathical changes in ZDF rats, and NO in the GN contributes to therapeutic effects of acupuncture on improvement in hyperalgesia and hypersensitivity in sensory diabetic neuropathy.

Neuropathy is a complication of diabetes mellitus with variable manifestations, and sensory aneuropathic pain is a complex, chronic pain state, most often accompanied by a tissue injury. Type II diabetes, non-insulin-dependent diabetes mellitus is becoming increasingly prevalent in many countries and affects 90% of diabetic patients. Recent studies have shown that pain and pressure thresholds are decreased in ZDF rats, a model of type II diabetes [6–8, 43]. Nerve conduction velocity is decreased in ZDF rats [3–5]. Previous pathological studies have shown that the large caliber dermal and small caliber epidermal axons in ZDF rats were lost [8], and the neuroaxonal dystrophy existed in the ileal mesenteric nerves of ZDF rats [7]. The present results support previous studies, which reported that pain and mechanical tolerance of the foot were decreased in ZDF rats. Our studies further show that decreased withdrawal latencies to application of mechanical, cold and thermal stimuli in ZDF rats were enhanced by EA ST36, which suggest that EA ST36 improves functional neuropathical changes in type II diabetic rats. Recent studies have demonstrated that high and low frequency EA effects are likely processed in different central areas [44, 45]. Low-frequency EA activates beta-endorphin and enkephalin systems, while high frequency EA activates dynorphin systems [45]. It has been demonstrated that EA ST36 with 10 Hz may be a better frequency to improve hyperalgesia and hypersensitivity in sensory diabetic neuropathy [46]. Our data are consistent with previous studies and demonstrate that EA ST36 with 10 and 30 Hz produced better therapeutic effects compared to EA with 3 and 60 Hz in a neuropathic rat model. The results suggest that EA ST36 with 10 Hz is an optimal frequency to treat sensory diabetic neuropathy.

GN receives ascending input from primary somatic sensory afferent fibers and the axons of dorsal horn neurons as illustrated in Figure 1. Peripheral somatosensory afferents from the hindlimb project to the GN [19–21] and neurons in the GN are activated by a single electrical stimulus to the sciatic nerve [19]. It has been demonstrated that the somatotopic organization of the GN receives peripheral somatosensory afferents from the hindlimb by electrophysiological mapping studies and anterograde axons tracing techniques in various mammals [20–24, 47, 48]. These neurons in the GN receive somatosensory afferent inputs originating from nociceptors and projecting to the thalamus [22, 47]. Several studies have shown that the GN is an integration centre for cutaneous and visceral information flowing into the thalamus, which plays an important role in somatic and visceral pain processing [22–24]. It has been pointed out that the activation of somatic afferents and acupuncture stimulation might modulate pain via somatosympathetic reflex [49, 50]. This finding has been verified through clinical studies with patients suffering from various kinds of chronic pain including diabetic neuropathic pain. Present studies show that EA ST36 increases NO release in the GN, which matches the time interval of improving withdrawal latencies to mechanical, cold and thermal nociception stimuli. Our result supports previous studies which reported that the GN is an integration centre for pain processes, and further demonstrates that NO is released in the GN following EA stimulation. Enhanced NO release in the GN is involved with the effects of EA ST36 on improvement in sensory diabetic neuropathy.

Recent studies have demonstrated that NO is involved in the nociceptive modulation, which contributes to analgesic mechanisms [51, 52]. Previous studies from this lab have demonstrated that expression of NO synthase in the GN is induced by sciatic nerve injury [27] and EA stimulation of hindlimb acupoints in rats [24–26]. Other investigators reported that impaired neuronal NO generation in diabetic rats induced hyperalgesia [30, 31]. The present results show that NO releases in the GN were markedly increased during EA ST36 in ZDF rats and moderately increased at 20–40 min after EA ST36 in LC rats. A large amount of NO is released in the GN immediately following EA stimulation in ZDF rats as compared with only a moderate release 20–40 min after EA in LC rats. This may reflect a somatosensory hypersensitivity of EA stimuli in neuropathic rats. Since somatosensory response to needle stimuli is higher in ZDF rats than that of LC rats, greater afferent inputs resulted in markedly increased dialysate NO_*x*_^*−*^ releases in the GN during EA ST36 in ZDF rats, but not in LC rats. In addition, the withdrawal latencies to mechanical, cold and thermal stimuli were improved by EA ST36 in both ZDF and LC rats. These withdrawal latencies to EA ST36 were further potentiated by infusion of an NO donor to mimic NO release and inhibited by microinfusion of an inhibitor of neuronal NO synthesis in the GN. The results consistently suggest that NO release in the GN is up-regulated by EA applied to ST36, and NO in the nucleus produces an inhibitory modulation of somatosensory/nociceptive susceptibility.

In summary, these results show that neuropathy occurs in ZDF rats with hyperalgesia and hypersensitivity to mechanical, thermal and cold stimuli in the hind foot. The withdrawal latencies were improved by EA ST36, but not altered by EA non-point. EA stimulation of ST36 with 10 and 30 Hz produced better therapeutic effects compared to EA with 3 and 60 Hz. Baseline NO release was lower in the GN of ZDF diabetic rats compared to that of LC rats. NO release in the GN was increased following EA ST36, and the therapeutic responses to EA ST36 in ZDF rats were further potentiated by the presence of an NO donor and inhibited by an inhibitor of NO synthesis in the GN. We conclude that endogenous NO release in the GN is decreased in ZDF rats with hyperalgesia and hypersensitivity of temperature and pressure. EA ST36-induced NO release in the GN contributes to its effects on improvement in sensory neuropathies in type II diabetic rats.

## Funding

Grant number AT004620 and AT002478 from the National Center for Complementary & Alternative Medicine (NCCAM), and Research Award (ADA 7-07-RA-100) from the American Diabetes Association (to S.-X. Ma).
